# Comprehensive Analysis and Characterization of the GATA Gene Family, with Emphasis on the GATA6 Transcription Factor in Poplar

**DOI:** 10.3390/ijms241814118

**Published:** 2023-09-14

**Authors:** Kai Zhao, Siyuan Nan, Yajing Li, Changhong Yu, Lieding Zhou, Jia Hu, Xia Jin, Youzhi Han, Shengji Wang

**Affiliations:** College of Forestry, Shanxi Agricultural University, Jinzhong 030801, China

**Keywords:** poplar, GATA family, tissue-differential expression, salt stress, co-expression analysis

## Abstract

GATA transcription factors are ubiquitously present in eukaryotic organisms and play a crucial role in multiple biological processes, such as plant growth, stress response, and hormone signaling. However, the study of GATA factors in poplar is currently limited to a small number of proteins, despite their evident functional importance. In this investigation, we utilized the most recent genome annotation and stringent criteria to identify 38 GATA transcription factor genes in poplar. Subsequently, we conducted a comprehensive analysis of this gene family, encompassing phylogenetic classification, protein characterization, analysis of promoter cis-acting elements, and determination of chromosomal location. Our examination of gene duplication events indicated that both tandem and segmental duplications have contributed to the expansion of the GATA gene family in poplar, with segmental duplication potentially being a major driving force. By performing collinearity analysis of genes across six different species, we identified 74 pairs of co-linear genes, which provide valuable insights for predicting gene functions from a comparative genomics perspective. Furthermore, through the analysis of gene expression patterns, we identified five GATA genes that exhibited differential expression in leaf–stem–root tissues and eight genes that were responsive to salt stress. Of particular interest was *GATA6*, which displayed strong induction by salt stress and overlapped between the two gene sets. We discovered that *GATA6* encodes a nuclear-localized protein with transcription activation activity, which is continuously induced by salt stress in leaf and root tissues. Moreover, we constructed a co-expression network centered around *GATA6*, suggesting the potential involvement of these genes in the growth, development, and response to abiotic stress processes in poplar through cell transport systems and protein modification mechanisms, such as vesicle-mediated transport, intracellular transport, ubiquitination, and deubiquitination. This research provides a foundation for further exploration of the functions and mechanisms of GATA transcription factors in poplar.

## 1. Introduction

Plant growth and development encompass a plethora of intricate biological processes, involving aspects such as tissue and organ differentiation, adaptive responses to environmental stimuli, and coping with various forms of stress. These processes require intricate and precise regulation and coordination of gene expression. Transcription factors, as pivotal gene regulatory factors, play a critical role in numerous essential biological processes. Within this category, the GATA family has emerged as a prominent group of transcription factors, exhibiting widespread participation in pivotal plant growth, development, and stress response mechanisms.

GATA transcription factors are widely present in eukaryotes, including animals, plants, and fungi, and are broadly involved in a variety of life activities. The first GATA factor was discovered during the process of erythroid-specific gene expression in vertebrates, and subsequently, the first plant GATA gene *NTL1*, which plays a key role in nitrogen metabolism, was cloned in tobacco [1,2,3]. GATA proteins, which are evolutionarily conserved regulatory factors, are named for their unique ability to recognize and bind to the T/AGATAA/G core sequences [4]. GATA proteins are characterized by conserved type-IV zinc finger motifs (C-X_2_-C-X_17-20_-C-X_2_-C), followed by a basic region [5]. The zinc finger establishes hydrophobic interactions with the minor groove of the target DNA [4]. Meanwhile, the basic stretch engages with the negatively charged phosphate backbone [4]. In plants, most GATA proteins contain a single C-X_2_-C-X_18_-C-X_2_-C zinc finger domain, with some members having 20 amino acid residue zinc finger loops or multiple zinc finger domains [6]. Their secondary structure is composed of four β-sheets and one α-helix [6]. Compared to animals, there are more GATA proteins in plants [4], and studies suggest that whole-genome duplications and dispersed duplication play a crucial role in the expansion of the GATA gene family [7]. By considering key features, plant GATA proteins can be categorized into four principal classes [6]. Research in certain plants has indicated that different types of GATA proteins possess specific characteristics. For instance, members of the first, second (excluding those with four amino acid residues between the first and second Cys), and fourth classes all contain a C-X_2_-C-X_18_-C-X_2_-C zinc finger loop. On the other hand, the third class of proteins contains a C-X_2_-C-X_20_-C-X_2_-C zinc finger loop [8]. Furthermore, the CCT domain with unknown function and the TIFY domain associated with the biological clock and hormone response are specifically present in the third type of proteins, while the ASXH domain is specifically present in the fourth type of proteins [8,9]. This suggests that different types of GATA proteins may possess specific functions. 

Initially, researchers focused on the key role of plant GATA transcription factors in regulating circadian rhythms and nitrogen metabolism [4,10]. As the biological functions of different GATA genes are being unraveled, it has been discovered that they are widely involved in important life processes such as plant growth and development, stress response, and hormone signal transduction [7]. GATA proteins are closely associated with various aspects of plant biology, including seed dormancy and germination, embryo development, shoot apical meristem development, chloroplast development, organ differentiation, flowering, hypocotyl and petiole elongation, leaf senescence, photosynthesis and growth, plant architecture, and yield. GATA2 in *Arabidopsis thaliana* mediates its photomorphogenesis process and plays a crucial role in light signal transduction [11]. Expression of *AtGATA12* is negatively regulated by gibberellin (GA) and can promote seed dormancy [12], while BME3 (AT3G54810) positively regulates the seed germination process [13]. HAN (AT3G50870) plays a crucial role in regulating the process of shoot apical meristem and flower development in Arabidopsis. Additionally, it is actively involved in controlling both cell proliferation and differentiation [14]. Overexpression of the *ZIM* (AT4G24470) in Arabidopsis has been shown to trigger significant elongation of both the hypocotyl and petiole [15]. Mutation of the *GNC* (AT5G56860) in Arabidopsis results in reduced chlorophyll levels and increased sensitivity to exogenous glucose [16]. *PdGNC*, an ortholog of *PtrGATA19*, has been identified to regulate chloroplast development and enhance plant photosynthetic capacity and growth under low nitrate conditions [17]. Further genetic transformation and functional analysis results indicate that overexpression of this gene significantly increases chlorophyll content, photosynthetic rate, plant height, growth rate, and biomass accumulation in transgenic poplar. In contrast, mutant plants exhibit severe growth retardation and enhanced secondary wood formation [18]. The Arabidopsis GNC and GNL (AT4G26150) inhibit flowering by directly repressing the expression of the flowering time regulator *SOC1* [19]. Overexpression of *OsGATA12* in rice leads to increased leaf greenness and reduced leaf and tiller numbers, and it affects yield parameters [20]. GATA proteins also play important roles in plant resistance to biotic and abiotic stresses. In Arabidopsis, heterologous expression of grape *VdGATA2* can prevent oxidative damage in plants and enhance their resistance against powdery mildew [21]. Analysis of cis-regulatory-element-mediated combinatorial regulation in rice infected with fungi reveals that complex regulatory cross-talks among transcription factor families such as GATA, WRKY, and MYB play crucial roles in plant defense responses [22]. PdGNC can be strongly induced by abscisic acid (ABA) and dehydration, enhancing water use efficiency by reducing stomatal aperture and increasing tolerance to water deficit in poplar [23]. The drought-stress-inducible gene *SlGATA17* enhances the drought resistance of tomatoes by regulating the activity of the phenylpropanoid biosynthetic pathway [24]. *OsGATA8* is induced by salt, drought, and ABA, and it improves plants’ tolerance to abiotic stress by regulating the expression of key genes involved in stress tolerance, reactive oxygen species scavenging, and chlorophyll biosynthesis [25]. The expression of *SlGATA17* is inhibited by NaCl treatment, and it negatively regulates the salt tolerance of tomatoes [26]. Sweet potato IbGATA24 can interact with IbCOP9-5a, thereby positively regulating plant tolerance to drought and salt [27]. In addition, GATA proteins are also involved in many important hormone signaling pathways. GNC and GNL simultaneously function as key transcriptional targets downstream of the GA signaling factors DELLA and PIFs, as well as the auxin pathway regulatory factors ARF2 and SLR [28,29]. They integrate the auxin and GA signals to control plant growth. In addition, studies have shown that GNC and GNL jointly control the emergence, greening, flowering time, and senescence of plants under the signaling pathways of auxins, cytokinins, GAs, and light [30]. Arabidopsis GATA2 mediates crosstalk between the brassinosteroid and the light signaling pathway, thereby regulating the photomorphogenesis process in plants [11]. Proteomic analysis results indicate that GATAs can interact with JAZ, a key regulator in the jasmonic acid (JA) signaling pathway [31].

GATA transcription factors play a crucial role in the life processes of plants. However, research on the proteins in poplar is limited, with only a few proteins’ functions and regulatory mechanisms being analyzed. Currently, the most in-depth research in this area focuses on the GNC protein in poplar. This protein plays a key role not only in photosynthesis, nitrogen utilization efficiency, and growth and development of poplar but also in mediating stomatal closure processes, thereby enhancing the drought resistance of poplar [17,18,23,32]. PdeGATA3 can regulate the expression of *PdeSTM* and alter the content of GA, leading to dwarfism in poplar [33]. *PtrGATA12* is mainly expressed in developing xylem tissues and plays an important role in regulating the biosynthesis of secondary cell wall components in poplar [34]. From this, it can be seen that GATA proteins are crucial in the growth, development, and defense against abiotic stress in poplar. The present investigation involved the identification of a total of 38 GATA proteins, leveraging the most recent poplar genome data. Following this, a systematic and comprehensive analysis of the gene family was carried out. Five key differentially expressed genes (DEGs) with tissue-differential expression patterns and eight genes responsive to salt stress in leaves were identified. We focused on *GATA6*, which showed strong induction under salt stress and differential expression patterns in root, stem, and leaf tissues, for further analysis. *GATA6* was found to encode a transcription factor localized in the nucleus with transcription activation activity, and it responded to salt stress in both leaf and root tissues of poplar. A gene co-expression network centered on *GATA6* was constructed, and GO enrichment analysis was performed on the gene set. The results indicated that *GATA6* and its co-regulators are likely to be involved in poplar growth, development, and abiotic stress responses through cell transport systems and protein modification mechanisms. These research findings provide a basis for deciphering the regulatory mechanisms of GATA proteins in poplar growth and development, exploring the stress adaptation mechanisms involving GATA proteins, and offering scientific evidence for genetic improvement of poplar.

## 2. Results

### 2.1. Identification and Phylogenetic Analysis of GATA Family Members in Poplar

In order to identify the GATA family members in poplar, we performed a search of the entire protein sequences of poplar using the GATA zinc finger (PF00320) and HMMER analysis (E-value < 1 × 10^−10^). The analysis revealed a total of 38 proteins identified as candidate proteins. Subsequently, we validated the candidate proteins using the InterPro and SMART databases [35,36]. The results showed that all 38 candidate proteins contained highly conserved GATA domains, which are characteristic of GATA transcription factor family members, and they were identified as GATA transcription factors in poplar. Based on the genomic positions of these genes, they were named *PtrGATA1*-*PtrGATA38* sequentially (Supplementary Table S1).

**Figure 1:**
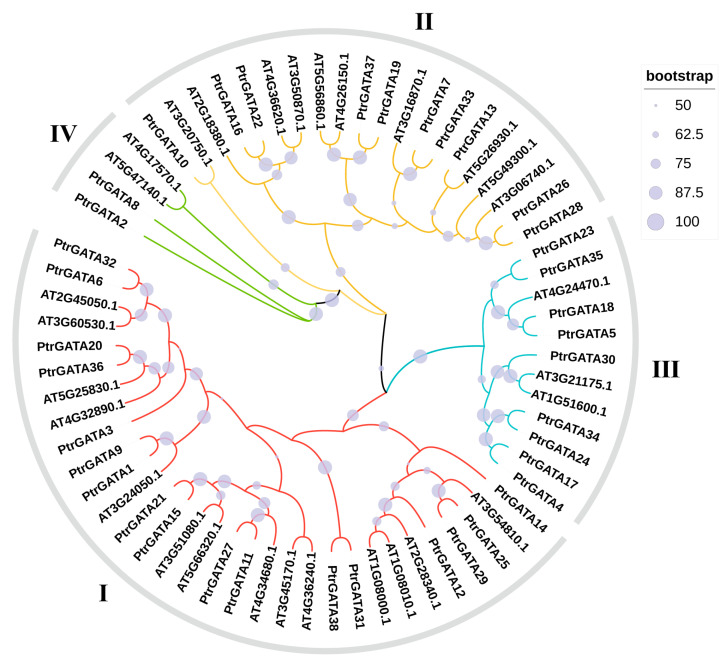
Dendrogram of GATA proteins in poplar and Arabidopsis. The dendrogram were constructed using the ML method with 1000 bootstrap tests. The colors (red, blue, yellow, and green) annotated on the branches represent different classes of GATA proteins.

In order to explore the evolutionary relationship and classify the GATA proteins in poplar, we constructed maximum likelihood (ML) phylogenetic trees using the amino acid sequences of GATA proteins from Arabidopsis and poplar. From Figure 1, it can be observed that the 38 GATA proteins in poplar were divided into four groups. The first group had the highest number of members (17), followed by the second and third groups with 10 and 9 members, respectively. The fourth group had the fewest members, consisting of only two proteins.

### 2.2. Characteristics Analysis of GATA Proteins in Poplar

**Figure 2:**
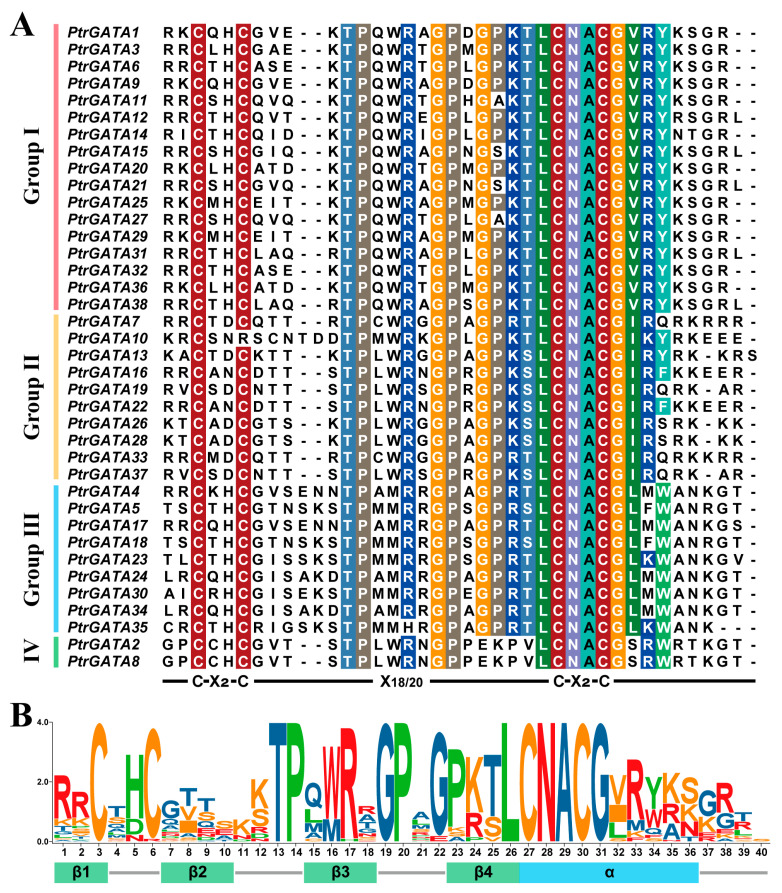
Visualization of the GATA DNA binding domain in poplar GATA proteins. (**A**) Multiple sequence alignment of the conserved GATA domain sequences. Different colors shade identical or similar amino acids at this position. Sorted according to the grouping in the phylogenetic tree. (**B**) Logo plot and secondary structure annotation of the conserved GATA domain sequences.

In order to clarify the characteristics of GATA proteins in poplar, we first extracted their conserved domain sequences and visualized them (Figure 2). Similar to what has been found in other plants, the conserved domain of GATA proteins in poplar also contains a type IV zinc finger motif, with a consensus sequence of C-X_2_-C-X_18/20_-C-X_2_-C. The first, second, and fourth classes of proteins share a common motif (C-X_2_-C-X_18_-C-X_2_-C), while the third class of proteins specifically possess a C-X_2_-C-X_20_-C-X_2_-C structure (Figure 2A). It is worth noting that besides the highly conserved cysteine residues, there are also many highly conserved amino acid residues in this domain, which may be related to the functions of GATA proteins in poplar, such as the preference for recognition of cis-acting elements. Furthermore, we found that certain amino acids at specific sites have undergone specific changes in different groups, which may be related to the functional differentiation of this protein class (Figure 2A). Consistent with studies in Arabidopsis [5], we also identified four β sheets and one α helix in the GATA domain of poplar (Figure 2B).

Further analysis of the physicochemical properties of the proteins predicted that there were differences among the 38 members of the poplar GATA transcription factor in terms of amino acid sequence length, protein molecular weight, theoretical isoelectric point, etc. (Supplementary Table S1). The average length of these proteins was 295.55 aa (ranging from 109 to 544 aa), and the average molecular weight was 32,745.43 Da. Additionally, among the protein groups, the fourth group had the largest protein length and molecular weight, despite having the fewest members (542 aa and 60,286.07 Da). The third and first groups followed with protein lengths and molecular weights of 311.56 aa and 34,202.67 Da, and 310 aa and 34,460.38 Da, respectively. The second group had the smallest protein length and molecular weight (207.3 aa and 23,010.36 Da). The theoretical isoelectric points of the poplar GATA proteins ranged from 4.86 to 10.69. The second group of proteins had smaller variations and larger theoretical isoelectric points, all above 7 (ranging from 7.95 to 9.78). In fact, 8 out of the top 10 proteins with the highest theoretical isoelectric points belonged to the second group. The third group of proteins exhibited comparatively lower theoretical isoelectric points and greater variations, wherein the protein with the highest theoretical isoelectric point and the five proteins displaying the smallest theoretical isoelectric points were all found within this particular group. The first group of proteins also had a large variation in theoretical isoelectric points (ranging from 5.65 to 9.06). The instability index (II) of these proteins ranged from 29.88 to 68.69. Only three proteins (PtrGATA10, PtrGATA23, and PtrGATA30) had an index below 40 and were predicted to be stable proteins, while the remaining 35 proteins were predicted to be unstable. There was also a significant difference in the aliphatic index of these proteins, ranging from 35.48 to 84.95. The grand average of hydropathicity for all proteins was less than 0, indicating that these proteins were all hydrophilic. Subcellular localization prediction results indicated that most of the poplar GATA proteins were predicted to be localized in the cell nucleus (33 in total), while three proteins (PtrGATA2, PtrGATA8, and PtrGATA21) were predicted to be localized in the chloroplast, PtrGATA23 was predicted to be extracellular, and PtrGATA35 was predicted to be cytoplasm.

### 2.3. Analysis of Cis-Acting Elements and Sequence Structures

There are numerous cis-acting elements on gene promoter sequences that have been proven to play a crucial role in gene transcription regulation and functional implementation. In order to clarify the distribution of cis-acting elements on the GATA gene promoter of poplar and predict their possible involvement in biological processes, the promoter sequences of each gene were extracted and analyzed, spanning 2000 bp. It is worth noting that a considerable magnitude of hormone- and stress-responsive elements are distributed on the promoter of this gene family, such as auxin-responsive element, GA-responsive element, ABA-responsive element, MeJA-responsive element, defense- and stress-responsive element, low-temperature-responsive element, drought-responsive element, wound-responsive element, and light-responsive element. In addition, there are also elements related to growth and development, such as element involved in circadian control, element involved in cell cycle regulation, element involved in seed-specific regulation, and element related to meristem expression (Supplementary Data S1). The above results indicate that poplar GATA transcription factors may widely participate in various biological processes, including poplar growth and development, as well as responses to adverse conditions, as well as play a key role in the life process of poplar. In addition, a large number of transcription factor binding sites are distributed on the promoters of *PtrGATAs* (Supplementary Data S1), indicating that these genes have diverse and complex functions and regulatory mechanisms.

**Figure 3:**
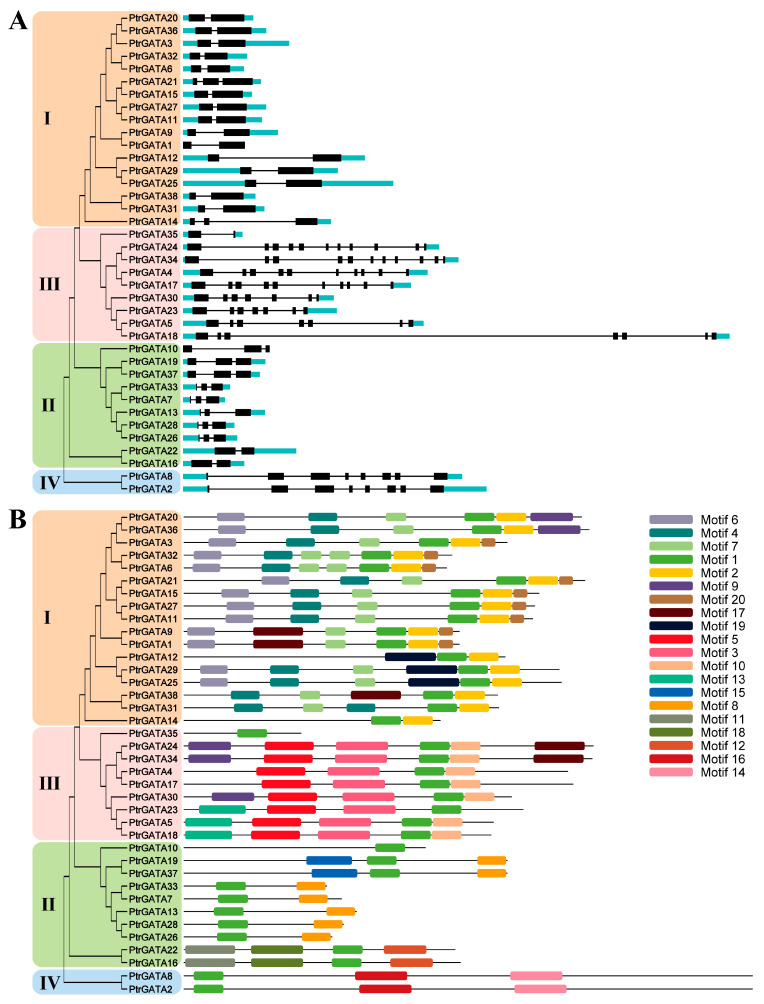
DNA structure and protein motif composition of poplar GATA family members. (**A**) DNA sequence structure. Cyan and black bars represent UTR regions and exons, respectively; black lines represent introns. (**B**) Protein motif distribution. Different colored squares represent different motifs. Sorted and classified based on the results of the phylogenetic tree.

To explore the gene and protein sequence structures of poplar GATA family members, visualization of their intron/exon structures and motif compositions was performed. Gene structure analysis revealed that most poplar GATA genes have UTR structures, except for *PtrGATA1* and *PtrGATA10*. Overall, there is significant variation in gene structures among different categories. Proteins with close phylogenetic relationships exhibit similar gene structures, such as 15 out of 17 members of the first group of GATA genes having two exons and one intron. In the second group, 8 out of 10 genes have three exons and two introns, while the other two members in a separate branch of the phylogenetic tree have two exons and one intron. The fourth group of genes has eight exons and seven introns. The genes in the third group show significant differences and can be divided into four types, but genes in the same branch of the phylogenetic tree have consistent structures (Figure 3A). Analysis of the motif composition of PtrGATAs identified a total of 20 conserved motifs (Figure 3B). Upon annotation of these motifs, it was found that most motifs had no significant annotation, except for motif 1, which was annotated as the GATA domain, motif 3 as the CCT motif, motif 5 as the TIFY domain, and motif 14 as the UCH-binding domain (Supplementary Table S2). Consistent with the expected results, all proteins contained motif 1, annotated as the GATA domain. Motif 2 was specifically present in all proteins of the first group, while motifs 3, 5, 10, and 13 were specifically present in proteins of the third group. Motifs 4, 6, 7, 19, and 20 were specifically present in proteins of the first group, while motifs 8, 11, 12, 15, and 18 were specifically present in proteins of the second group. Motifs 9 and 17 were present in a few proteins of the first and third groups, while motifs 14 and 16 were specifically present in proteins of the fourth group (Figure 3B). The specific distribution of numerous motifs may be related to the functional differentiation of proteins in each group.

### 2.4. Chromosomal Localization and Collinearity Analysis

**Figure 4:**
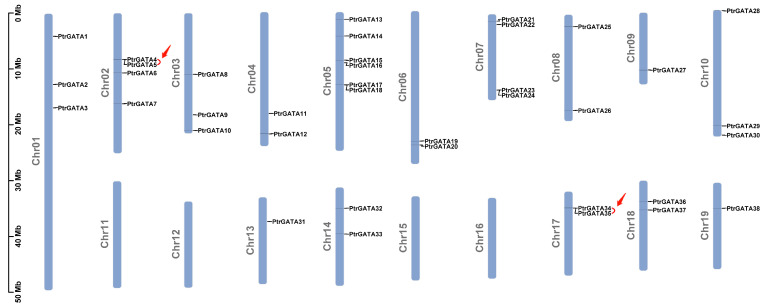
Chromosomal localization of poplar GATA genes and analysis of tandem duplication events between genes. The red arrows and curves indicate the gene pairs that have experienced tandem duplication events.

By extracting and visualizing the annotation information of the poplar genome, 38 GATA genes were located on 15 out of 19 chromosomes, excluding chromosomes 11, 12, 15, and 16 (Figure 4). In addition, it can be observed that the distribution of these genes on the chromosomes is not uniform and is not correlated with the length of the chromosomes.

**Figure 5:**
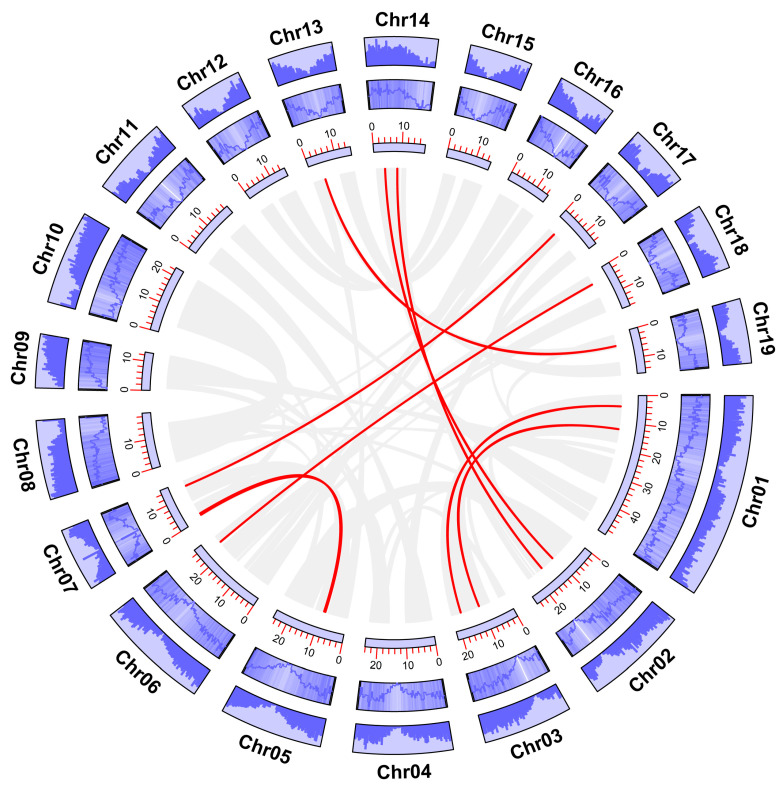
Identification of segmental duplication events between poplar GATA genes. Purple dashed lines, heat maps, and bar charts represent gene density on different chromosomes in poplar. Gray lines indicate segmental duplication events in the poplar genome, while red lines highlight GATA gene pairs that have undergone segmental duplication events.

The expansion of gene families is closely related to the occurrence of gene duplication events. Therefore, the duplication events in the poplar GATA gene family were identified. The analysis results showed that there were two pairs of GATA genes that underwent tandem duplication events, located on chromosomes 02 and 17 (Figure 4). In addition, there were 10 pairs of GATA genes that underwent segmental duplication events, distributed on 11 chromosomes of poplar (Figure 5, Supplementary Table S3). These results indicate that both tandem and segmental duplication events play a role in the expansion of the poplar GATA gene family, and segmental duplication events may be one of the main driving forces behind this phenomenon.

**Figure 6:**
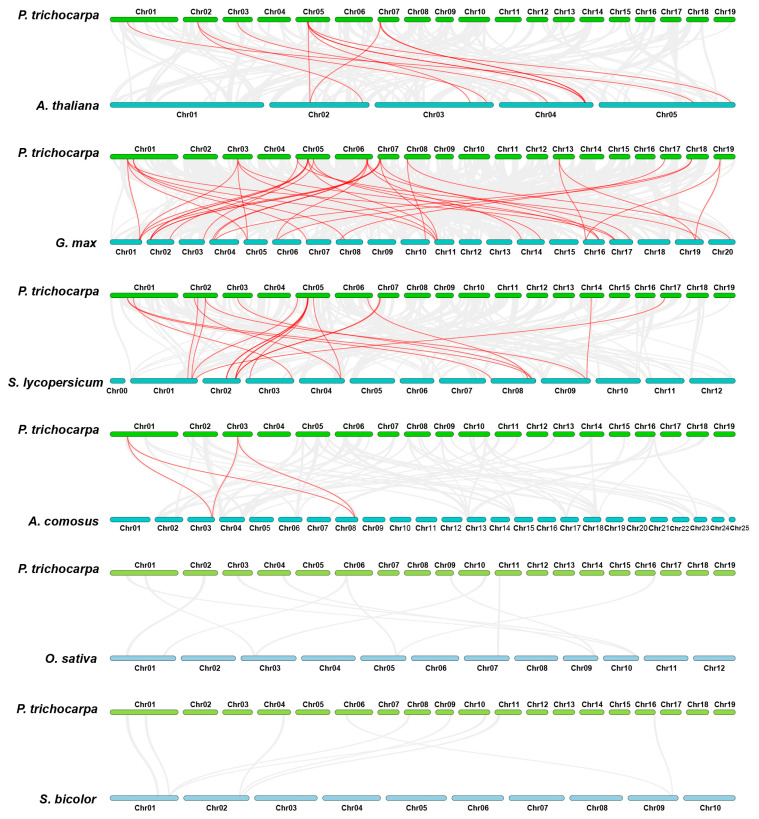
Gene collinearity analysis among species. Gray lines indicate all collinear blocks between species, while red lines highlight collinear gene pairs associated with poplar GATA genes.

In addition, we conducted gene collinearity analysis among different species. Six representative genomes were used for comparative analysis with the poplar genes, including three dicotyledonous plants, namely, *Arabidopsis thaliana*, *Glycine max*, and *Solanum lycopersicum*, and three monocotyledonous plants, namely, *Oryza sativa*, *Sorghum bicolor*, and *Ananas comosus*. The results showed that 21 poplar GATA genes exhibited collinearity with 9 Arabidopsis genes, 25 soybean genes, 14 tomato genes, and 2 pineapple genes, but did not show collinearity with rice and sorghum genes (Figure 6, Supplementary Table S4). In total, 74 pairs of collinear genes were identified in the six comparisons. Consistent with studies in other gene families [37], poplar GATA genes showed a higher degree of collinearity with soybean, tomato, and Arabidopsis genes, which are also dicotyledonous plants, with 38, 20, and 12 gene pairs, respectively. However, there were fewer collinear gene pairs between poplar GATA genes and monocotyledonous plants, with only four gene pairs with pineapple and no collinearity with rice and sorghum. Further analysis revealed that a significant quantity of GATA genes showed collinearity with multiple genes in the same species, especially between poplar and soybean. Among the 16 poplar GATA genes, 15 genes showed collinearity with multiple soybean genes, particularly *PtrGATA2* and *PtrGATA8*, which showed collinearity with four identical soybean genes each. Interestingly, in the comparison between poplar and pineapple, only *PtrGATA2* and *PtrGATA8* showed collinearity with two identical pineapple genes. Considering that *PtrGATA2* and *PtrGATA8* are the only members of the fourth group in the poplar GATA gene family, these results suggest that this group of proteins may have relatively conserved gene functions.

To further investigate the evolutionary constraints among GATA genes, the non-synonymous (Ka)/synonymous (Ks) ratios were calculated for the identified tandem duplicates, segmental duplicates, and collinear gene pairs. The results showed that, except for some gene pairs where the Ks values were not available for calculation, the Ka/Ks ratios for all other gene pairs were less than 1 (Supplementary Tables S3 and S4), indicating strong purifying selection pressure during the evolution of the GATA gene family.

### 2.5. Tissue-Differential Expression of GATA Genes in Poplar

**Figure 7:**
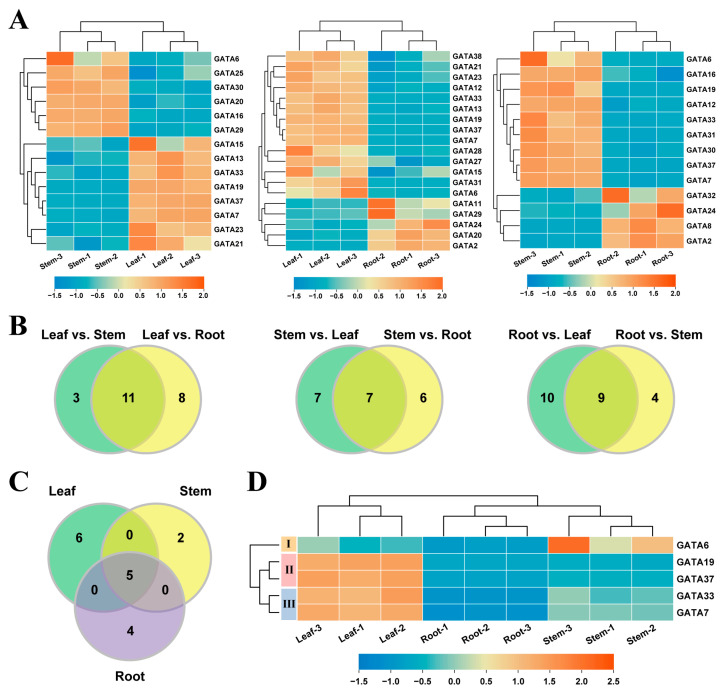
Analysis of tissue-differential expression of poplar GATA genes. (**A**) Heatmap of gene expression of DEGs in different tissue pairs. (**B**) Venn diagram of DEGs between different tissue pairs. The number of shared genes in the figure represents genes that are differentially expressed in one tissue compared to the other two tissues. (**C**) Venn diagram of three groups of shared genes in panel (**B**). The shared genes in this figure represent genes that are differentially expressed between any two tissues out of the root, stem, and leaf. (**D**) Heatmap of gene expression of shared genes in panel (**C**). All heatmaps are generated using gene expression FPKM values.

To explore the expression patterns of GATA genes in different tissues of poplar, the gene expression levels in roots, stems, and leaves were analyzed. By comparing the gene expression in different tissue pairs, 14, 19, and 13 DEGs were identified in the leaf–stem, leaf–root, and stem–root tissue pairs, respectively (Figure 7A, Supplementary Data S2). In the leaf–stem tissue comparison, eight GATA genes were found to be upregulated in the leaf and six genes were downregulated. In the leaf–root tissue comparison, 14 GATA genes were upregulated in the leaf and 5 genes were downregulated. In the stem–root tissue comparison, nine genes were upregulated in the stem and four genes were downregulated. Based on these results, we compared the DEGs in different tissue pairs and identified genes that showed tissue-specific differential expression (Figure 7B). The results showed that compared to the stem and root tissues, 11 genes exhibited differential expression in the leaf; compared to the leaf and root tissues, 7 genes exhibited differential expression in the stem; compared to the leaf and stem tissues, 9 genes exhibited differential expression in the root. Further analysis revealed that among these three sets of genes, there were five genes shared by all of them (Figure 7C). These genes showed differential expression in any two of the leaf, stem, and root tissues. By analyzing the expression patterns of these five genes, they were divided into three groups. The first group of genes were progressively downregulated in the stem, leaf, and root, sequentially. The second and third groups of genes were progressively downregulated in the leaf, stem, and root, sequentially, but there were differences in the expression levels of the genes.

### 2.6. Analysis of Salt Stress Response of GATA Genes in Poplar

**Figure 8:**
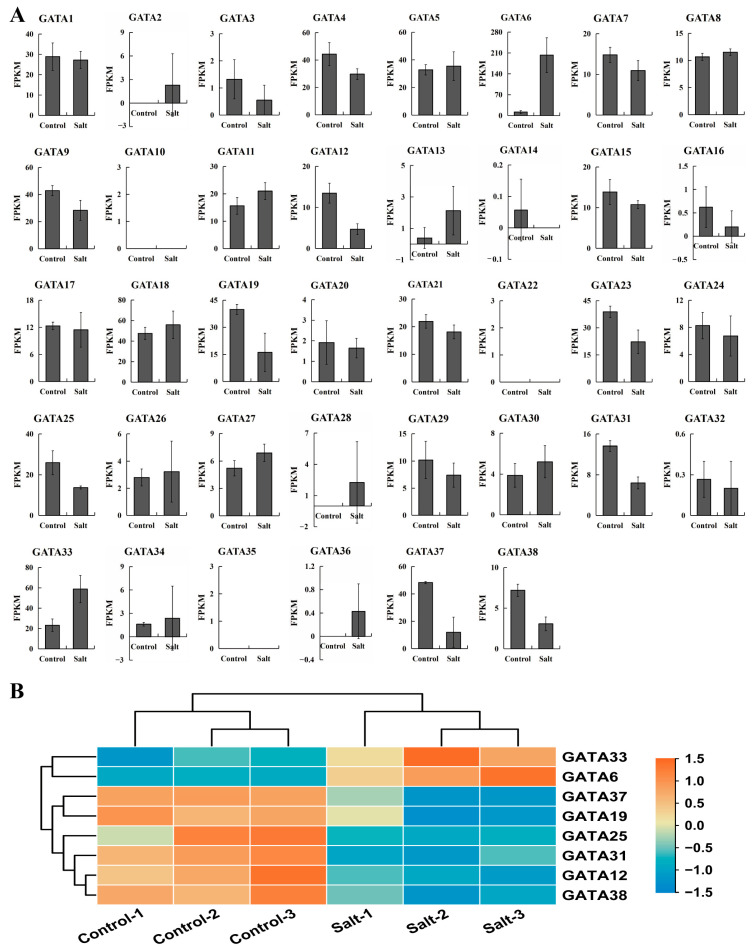
The response of poplar GATA genes to salt stress. (**A**) Expression levels of GATA genes in poplar leaves before and after salt stress. (**B**) Heatmap of DEGs in poplar leaves in response to salt stress. Gene expression FPKM values were used for plotting.

In order to further investigate the response of GATA genes in poplar to salt stress, the expression patterns were analyzed using transcriptome sequencing data (Figure 8, Supplementary Data S3). As shown in Figure 8A, the expression levels of most GATA genes did not change significantly before and after salt stress. Subsequently, we used DESeq2 to screen for DEGs and identified a total of eight genes that responded to salt stress, including two upregulated genes and six downregulated genes (Figure 8B). Analysis of the degree of differential expression of these genes revealed that the downregulated genes showed a moderate decrease, with four out of six genes showing a 2–3-fold decrease, and the lowest downregulation being 4.38-fold. Among the upregulated genes, *GATA33* was only upregulated 2.31-fold, while *GATA6* showed a higher level of upregulation, reaching 15.48-fold (Supplementary Data S3).

### 2.7. Subcellular Localization and Transcriptional Activation Activity Analysis of Poplar GATA6

**Figure 9:**
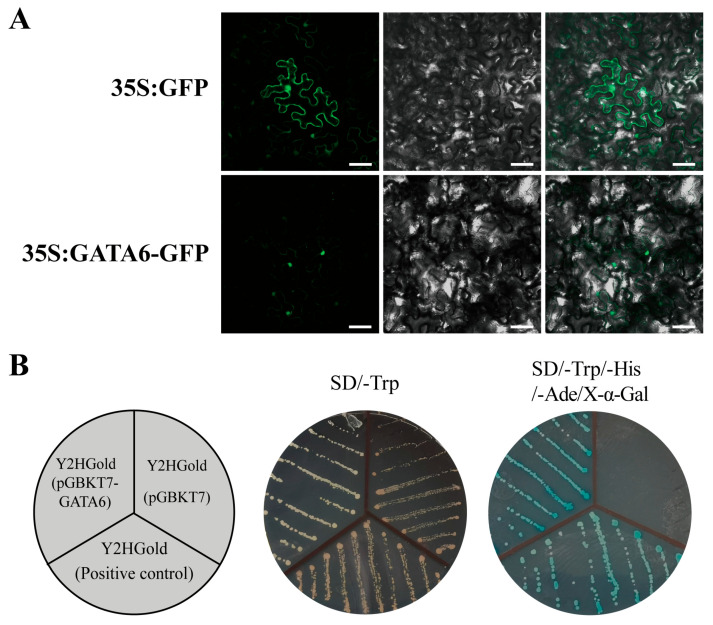
Subcellular localization and transcriptional activation activity analysis of poplar GATA6. (**A**) Subcellular localization. From left to right: GFP fluorescence, bright-field, and merged images of GFP fluorescence and bright-field. GFP empty vector was used as control. Scale bar = 40 μm. (**B**) Transcriptional activation activity analysis. Yeast cells transformed with pGBKT7 empty vector or pGBKT7-53/pGADT7-T vectors were used as negative or positive controls, respectively.

*GATA6* exhibits significant responsiveness to salt stress in poplar leaves, displaying the highest degree of upregulation. Furthermore, this gene demonstrates a distinctive expression pattern in various tissues of poplar, such as the root, stem, and leaf, implying its potential pivotal role in the growth, development, and response to salt stress in poplar. Consequently, further investigations concerning this gene are warranted. To explore the subcellular localization of GATA6, an agrobacterium-mediated transient expression experiment was conducted in tobacco leaves. The results, as depicted in Figure 9A, reveal that the fluorescence of the GATA6-GFP fusion protein was exclusively observed within the nucleus, while the fluorescence of the control protein was distributed throughout the cell. These outcomes unequivocally confirm that GATA6 is a transcription factor localized within the nucleus in poplar.

To determine whether GATA6 has transcriptional activation activity, a yeast assay was performed for analysis. As shown in Figure 9B, yeast cells transformed with the pGBKT7-GATA6 vector, negative control, and positive control were able to grow on solid SD/-Trp medium. However, only yeast cells transformed with the pGBKT7-GATA6 vector and the positive control were able to grow and turn blue on solid SD/-Trp/-His/-Ade/X-α-Gal medium. These results indicate that the GATA6 transcription factor in poplar possesses transcriptional activation activity.

### 2.8. Analysis of the Spatiotemporal Expression Pattern of the Poplar GATA6

**Figure 10:**
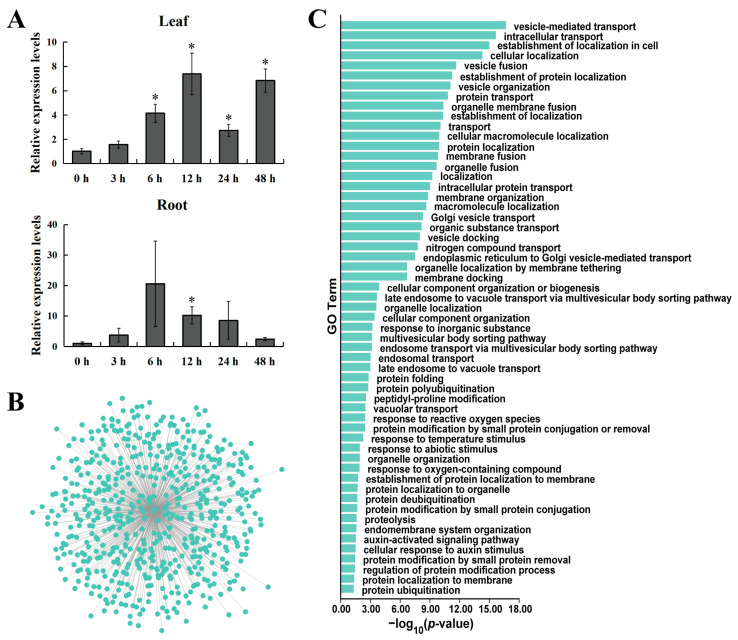
Gene spatiotemporal expression patterns and co-expression network analysis. (**A**) Relative expression of *GATA6* at different time points under salt stress in poplar leaves and roots. The expression levels in untreated tissues were used as reference for calculation. Asterisks (*) indicate significant differences between treated and control samples (*t*-test, *p* < 0.05). (**B**) Co-expression network centered around *GATA6*. (**C**) GO enrichment analysis of the co-expressed gene set.

In order to investigate the spatiotemporal expression pattern of the *GATA6* gene in response to salt stress, RT-qPCR was used to analyze gene expression. The experimental results showed that at different time points under salt stress conditions, the *GATA6* overall showed an upregulated expression pattern in the leaf and root tissues of poplar, peaking at 12 h and 6 h, respectively (Figure 10A). These results suggest that *GATA6* is likely to be involved in the process of poplar response to salt stress.

### 2.9. Co-Expression Analysis of Genes and GO Enrichment Analysis

In order to explore genes co-expressed with *GATA6* and identify the biological processes they are involved in, a gene co-expression network centered around *GATA6* was constructed and a GO enrichment analysis was performed on the gene set. Using the Weighted Correlation Network Analysis (WGCNA) method, a total of 590 genes co-expressed with *GATA6* were identified (Figure 10B, Supplementary Data S4). GO enrichment analysis of this gene set revealed significant enrichment of *GATA6* and its co-expressed genes in 57 biological processes, including vesicle-mediated transport, intracellular transport, protein transport, Golgi vesicle transport, protein polyubiquitination, response to reactive oxygen species, response to temperature stimulus, response to abiotic stimulus, protein deubiquitination, proteolysis, auxin-activated signaling pathway, cellular response to auxin stimulus, regulation of protein modification process, and protein ubiquitination. These results suggest that these genes are likely involved in the growth, development, and response to abiotic stress in poplar through processes such as vesicle transport, intracellular transport, protein ubiquitination, and protein deubiquitination.

## 3. Discussion

Currently, the functions of many GATA proteins have been analyzed in Arabidopsis, but the research on GATA factors in woody model plants, such as poplar, is limited. Functional validation of GATA genes, such as *PdGNC*, *PdeGATA3*, and *PtrGATA12*, has been conducted in poplar and found to have a significant impact on the growth and development processes [17,18,23,32,33,34]. In particular, *PdGNC* not only mediates key life activities such as photosynthesis and nitrogen utilization efficiency but also plays a role in resistance to abiotic stress. These studies indicate that poplar GATA proteins are likely involved in many important biological processes. Hence, it is crucial to conduct a comprehensive identification and analysis of all members within this gene family, as well as to screen and identify key factors responsible for tissue-specific differences and stress responses. In this study, based on the latest poplar genome information and strict screening criteria, 38 GATA family members were identified from the poplar genome, named *PtrGATA1* to *PtrGATA38* according to their distribution on chromosomes. By comparing with the 39 proteins mentioned in previous study [34], it has come to light that alterations in the annotation information have occurred for 12 genes in the recently updated database. Among these, 11 genes have changes in their transcript ID, and one gene has an updated gene ID. Furthermore, a gene denoted as *Potri.T158300*, which was previously unassigned to a particular chromosome, has been removed from the updated database. Consequently, the revised database now encompasses a total of 38 GATA genes within the poplar genome. Protein feature analysis results show that, consistent with the features found in other plants, the GATA domain of poplar GATA proteins is also composed of four β-sheets and one α-helix, including a type IV zinc finger motif with a consensus sequence of C-X_2_-C-X_18/20_-C-X_2_-C (Figure 2). Many highly conserved amino acid residues in the domain are likely associated with the specific functions of this gene family.

Upstream regulatory factors can regulate the expression levels of target genes by recognizing and binding cis-acting elements on the promoter. This, in turn, regulates various biological processes. Currently, a large number of cis-acting elements and their associated functions or pathways have been deciphered. Through analysis of the promoter sequences of GATA genes in poplar, a remarkable number of hormone and stress response elements have been identified (Supplementary Data S1). Plant hormones are organic compounds produced in trace amounts in plants that regulate their physiological processes and play critical roles throughout their life cycle. Auxin is present in almost all stages of plant growth and development, playing a role not only in plant development and environmental adaptation but also as an important signaling molecule for communication between different tissues and cells [38]. GAs are involved in regulating various processes of plant growth and development, including seed germination; stem elongation; leaf expansion; pollen maturation; and flower, fruit, and seed development, as well as regulating plant adaptation to stress [39]. ABA is acknowledged for its robust ability to bolster resistance to stress, and it also plays a pivotal role in regulating various processes, including tillering, flowering, and seed development [40]. JA plays multiple regulatory roles in plant growth and development, including embryogenesis, flower development, leaf senescence, root formation, and stomatal opening. It can also enhance plant resistance under various adverse environmental conditions through the JA signaling pathway [41]. Numerous studies have shown that plant GATA genes play critical roles in the auxin, cytokinin, GA, JA, and brassinosteroid signaling pathways. Therefore, it is likely that poplar GATA genes mediate various processes of plant growth and development and their adaptation to the environment through these hormone signaling pathways.

Through identifying and annotating the motif composition of poplar GATA proteins, a total of 20 conserved motifs were found (Supplementary Table S2). Among these motifs, only four were annotated as GATA, CCT, TIFY, and UCH-binding domains, individually. Except for motif 1, which was annotated as the GATA domain, a large number of motifs are specifically distributed in certain protein classes, possibly related to the functional differentiation of this protein family. Consistent with studies in other plants, the CCT and TIFY motifs are specifically present in the third class of GATA proteins. The function of the CCT motif is not yet clear, but many proteins containing this motif are associated with integrating day length and rhythmicity [9], while the TIFY motif has been reported to be involved in the regulation of the biological clock and hormone signaling response [8]. Therefore, the third class of poplar GATA proteins is likely to be involved in the regulation of diurnal rhythms and hormone responses. Motif 14, which is annotated as the UCH-binding domain, may be associated with protein ubiquitination processes [42], and is specifically present in the fourth class of proteins. Additionally, motif 14 is also annotated as the Asx homology domain (ASXH), but with a relatively high E-value of 0.016. This domain is reported to be distributed in various transcription factors, coactivators, and corepressors, and may mediate their interactions [43].

Comparative analysis of GATA protein numbers in eukaryotes reveals that plants have a higher number of GATA proteins encoded in their genomes compared to other organisms. For example, humans, *Drosophila melanogaster*, and *Schizosaccharomyces pombe* have been found to have six, eight, and four GATA genes, respectively, while Arabidopsis and rice have 29 and 28 members, respectively [6]. Previous studies have suggested that whole-genome duplication plays a key role in the expansion of the GATA family [7]. Given the large number of GATA proteins encoded in the poplar genome, we have identified tandem and segmental duplication events that occur between genes. The analysis results show that there are two pairs of GATA genes involved in tandem duplication events, and ten pairs involved in segmental duplication events (Figure 4 and Figure 5, Supplementary Table S3). Therefore, both tandem and segmental duplication events play a role in the expansion of the poplar GATA gene family, with segmental duplication events being one of the main driving forces. In addition, we have further analyzed the collinearity relationship between poplar GATA genes and genes in other species. Similar to studies on other gene families, poplar GATA genes have a significant collinearity relationship with soybean, tomato, and Arabidopsis genes, which are also dicotyledonous plants, with 38, 20, and 12 gene pairs, respectively (Figure 6, Supplementary Table S4). There are fewer collinearity gene pairs with monocotyledonous plants, with only four gene pairs with pineapple and no gene pairs with rice and sorghum. It is worth noting that the only two members of the fourth class of poplar GATA genes, *PtrGATA2* and *PtrGATA8*, have collinearity relationships with four soybean genes and two pineapple genes, respectively, and these are the only two genes in poplar that have collinearity relationships with pineapple genes. These results suggest that the fourth class of poplar GATA proteins may have conserved functions. Furthermore, analogous to other land plant genomes, the fourth class of poplar GATA proteins is the smallest class in this family, indicating that selective pressure maintains a smaller number of copies of this subfamily. However, the underlying reasons for this still need further investigation. 

GATA genes hold significant significance in numerous essential mechanisms of plant growth, development, and the ability to withstand stress. Salt stress is considered a prominent abiotic stress factor that exerts a significant impact on various aspects of plant life, including growth, distribution, and yield. Saline–alkali soils are widely distributed around the world, and their high salt concentration inhibits plant growth and development, leading to leaf yellowing, plant withering, and root damage. The response of plants to salt stress is a complex biological process involving the regulation of various physiological and biochemical mechanisms. Investigating plant responses to salt stress allows for the elucidation of molecular mechanisms underlying plant adaptation to environmental stress, the identification of novel stress-resistant gene resources, and the enhancement of productivity in saline–alkali soils. Consequently, it is imperative to examine the distinct expression patterns of GATA genes across different tissues and their response to salt stress in poplar. By comparing the gene expression levels between different tissues, we identified 11, 7, and 9 tissue-differential DEGs in leaves, stems, and roots, respectively (Figure 7). Further comparative analysis identified five genes that showed differential expression between any two of the three tissues (leaves, stems, and roots). These genes may be involved in regulating key processes such as gene expression, signal transduction, and metabolic pathways in poplar growth and development. By analyzing the expression levels of GATA genes in leaf tissues before and after salt stress treatment, we identified eight salt-stress-responsive genes, including two upregulated and six downregulated genes (Figure 8). Among the two upregulated genes, *GATA6* showed a 15.48-fold upregulation, which is much higher than the 2.31-fold upregulation of *GATA33* (Supplementary Data S3). Furthermore, *GATA6* is also one of the five key genes identified with tissue-differential expression, suggesting that it has a broad and strong functionality. Therefore, further analysis of GATA6 is warranted.

Like many transcription factors, poplar GATA6 is a nuclear protein with transcriptional activation activity (Figure 9). In addition, the analysis of gene spatiotemporal expression patterns showed that this gene is upregulated in leaf and root tissues at five time points after salt stress (Figure 10A). To further explore the biological processes that *GATA6* may be involved in, we constructed a gene co-expression network centered around this gene and performed GO enrichment analysis on the genes involved (Figure 10B,C). The results indicate that these genes may participate in the growth, development, and abiotic stress response in poplar through processes such as vesicular transport, intracellular transport, protein ubiquitination, and protein deubiquitination. Plants exhibit a sophisticated endomembrane system that effectively employs diverse mechanisms for protein synthesis, processing, and transportation. Of particular importance is the vesicular transport pathway, which facilitates the movement of substances across distinct compartments within plant cells. This process holds considerable significance in the transportation of substances, regulation of hormones, and intercellular signaling within plant cells. Moreover, it serves as a pivotal player in the growth, development, and response to stress exhibited by plants [44,45]. Intracellular transport participates in regulating the distribution of substances and signal transduction in plants, ensuring normal growth and adaptation to environmental changes. Ubiquitination and deubiquitination are important physiological processes related to specific protein degradation in cells and are also important regulatory mechanisms in cellular signaling pathways. They are crucial for maintaining normal plant growth, development, and environmental adaptability. The ubiquitin–proteasome system is a rapid regulatory mechanism for selective protein degradation in plants and is responsible for the degradation of most cellular proteins, thus participating in the regulation of a wide range of cytological and physiological processes [46]. Interestingly, research has shown that autophagy, a part of the vesicular transport system, interacts with the ubiquitin–proteasome system [47]. Therefore, these important biological processes, in which poplar *GATA6* and its co-expressed genes are involved, can be interconnected into a complex regulatory network. It is worth noting that *AtGATA2* (*AT2G45050*), which shows collinearity with poplar *GATA6*, is negatively regulated by COP1-dependent ubiquitination and proteasomal degradation and can be ubiquitinated by COP1 in vitro [11]. *AtGATA2* functions as a mediator of crosstalk between the light and brassinosteroid signaling pathways, thereby contributing to the process of photomorphogenesis in Arabidopsis [11]. Specifically, in the root meristem, *AtGATA2* is regulated by auxin and exerts a regulatory role in limiting the activity of cell division [48]. These findings suggest that *GATA6* potentially plays various roles in regulating the growth and development processes of poplar.

## 4. Materials and Methods

### 4.1. Identification and Phylogenetic Analysis of GATA Family Members in Poplar

In order to identify the GATA family members in poplar, protein sequences of *Populus trichocarpa* (v4.1) were retrieved from the Phytozome v13 database [49,50]. The retrieved protein sequences were then aligned and searched against the GATA zinc finger domain (PF00320) using HMMER 3.0 with an E-value cutoff of 1 × 10^−10^ [51]. Candidate proteins were validated using the InterPro and SMART databases [35,36]. Finally, the GATA family proteins in poplar were identified and named according to their distribution order on the chromosomes.

The GATA protein members and grouping information of Arabidopsis were obtained from previous studies [6]. The amino acid sequences of the relevant proteins were downloaded from the Phytozome v13 database [49,50,52]. The amino acid sequences were imported into TBtools v2.003 software to construct a ML phylogenetic tree with 1000 bootstrap replicates [53]. The resulting phylogenetic tree was redrawn using iTOL v6 [54].

### 4.2. Characteristic Analysis of Poplar GATA Proteins

Visualization of the conserved domain sequences of poplar GATA proteins was performed using BioEdit v7.0.5.3 [55]. The sequences were sorted and classified based on the grouping information from the phylogenetic tree. ClustalX 1.83 was used to align the domain sequences [56], and the alignment results were visualized using TBtools v2.003 [53]. The physicochemical properties of the proteins were analyzed using ProtParam [57], and the subcellular localization of the proteins was predicted using WoLF PSORT [58].

### 4.3. Analysis of Cis-Acting Elements and Sequence Structure

The genomic, promoter, and coding sequences of the gene were downloaded from the Phytozome v13 database [49,50]. The sequence of 2000 bp upstream of the transcription start site of the poplar GATA gene was extracted as the promoter sequence, which was then analyzed using the PlantCARE and PlantPAN 4.0 databases to determine the distribution of cis-acting elements and obtain their descriptive information [59,60]. The gene structure diagram was generated using the Gene Structure Display Server 2.0 [61]. The presence of motifs on the protein was identified and visualized using MEME v5.5.4 and TBtools v2.003 [53,62]. The motifs were annotated using the Pfam and SMART databases [35,51].

### 4.4. Chromosome Localization and Collinearity Analysis

The genome annotation information of poplar was downloaded from the Phytozome v13 database [49,50]. TBtools v2.003 was used to extract and visualize the chromosomal localization information of poplar GATA genes [53]. Tandem duplication events between poplar GATA genes were identified using TBtools v2.003 with MCScanX [53,63]. TBtools v2.003 software, MCScanX, and BLASTP were used to analyze segmental duplication events between GATA genes and the collinearity relationships between genes in different species [53,63]. TBtools v2.003 was used to calculate the Ka and Ks ratios between gene pairs [53].

### 4.5. Gene Expression Pattern Analysis

Transcriptome sequencing technology was used to analyze the expression levels of poplar GATA genes. Leaf, stem, and root tissues of one-month-old tissue-cultured poplar seedlings were used for transcriptome sequencing. The cultivation conditions included an average temperature of 25 °C and a light–dark cycle of 16 h of light and 8 h of darkness. The sequencing method used was paired-end 150 bp, with a sequencing depth of more than 10 times and three biological replicates. The DEGs were screened using DESeq2 [64], with a threshold of fold change greater than or equal to twofold and a padj value less than or equal to 0.05. The expression levels of genes were normalized using FPKM values.

### 4.6. Subcellular Localization and Transcriptional Activation Analysis

Subcellular localization analysis was conducted using agrobacterium-mediated transient transformation in tobacco leaves. Firstly, gene cloning primers were designed based on the transcript sequence of the *GATA6* gene in poplar. The coding sequence of *GATA6* was amplified using poplar leaf cDNA as a template. Subsequently, the coding sequence of the *GATA6* gene without the stop codon was cloned into the pBI121-GFP vector, resulting in a fusion expression vector of GATA6 and GFP protein. Finally, the fusion expression vector was transferred into agrobacterium strain EHA105 and transiently expressed through injection into tobacco leaves. The GFP fluorescence was observed using a confocal laser scanning microscope.

Yeast experiments were conducted to analyze the transcriptional activation activity of the protein. The coding sequence of the poplar *GATA6* gene was cloned into the pGBKT7 yeast expression vector. After plasmid extraction, the plasmid was transformed into Y2Hgold yeast cells. The transformed Y2Hgold yeast cells containing different plasmids were streaked on SD/-Trp and SD/-Trp/-His/-Ade/X-α-Gal solid culture media. The growth of the cells was observed to determine whether the protein has transcriptional activation activity. Y2Hgold yeast cells transformed with the pGBKT7 empty vector were used as a negative control, and Y2Hgold yeast cells transformed with the pGBKT7-53/pGADT7-T vectors were used as a positive control. The primers used in this experiment are listed in Supplementary Table S5.

### 4.7. Analysis of Gene Spatiotemporal Expression Pattern

The spatiotemporal expression pattern of the gene under salt stress was analyzed using RT-qPCR. One-month-old tissue-cultured seedlings were taken out from the culture medium and continued to be cultured in soil pots for another month under the same conditions as mentioned earlier. The seedlings were subjected to salt stress by treating them with 150 mM NaCl solution for 3, 6, 12, 24, and 48 h, with water treatment serving as the control. Three biological replicates were set up for each treatment. After the treatment, all leaf and root samples were collected simultaneously. RNA was extracted from each sample and reverse transcribed into cDNA, followed by RT-qPCR experiments using pre-designed primers. The *Actin* gene was used as an internal reference gene, and the relative expression levels of the genes were calculated using the 2^−ΔΔCt^ method.

### 4.8. Co-Expression Analysis of Genes and GO Enrichment Analysis

A gene co-expression network centered around poplar *GATA6* was constructed using the WGCNA method and gene expression data from 15 RNA-Seq samples [65]. Cytoscape v3.5.1 was then employed to visualize the network [66]. The co-expression networks were mapped out by selecting the top 20% gene pairs that exhibited the strongest weights. TBtools v2.003 was used to perform GO enrichment analysis on the gene set, where we focused on the biological processes in which these genes are involved [53].

## 5. Conclusions

Based on the most recent genomic data of poplar and utilizing stringent criteria, this study successfully identified 38 members of the poplar GATA transcription factor family and conducted a comprehensive and systematic analysis. These poplar GATA proteins can be categorized into four classes, characterized by a conserved GATA domain that contains an IV-type zinc finger motif, with a consensus sequence of C-X_2_-C-X_18/20_-C-X_2_-C. Although these proteins exhibit significant differences in their physicochemical properties, they are all hydrophilic proteins. Most of these proteins are predicted to be located within the nucleus, while a few are found in the chloroplast, extracellular, or cytoplasmic regions. The presence of numerous hormone and stress-responsive cis-acting elements on their promoters suggests that these GATA proteins may be involved in various biological processes, such as growth, development, and stress response in poplar. Furthermore, each class of GATA proteins possesses specific motifs with unknown functions, indicating their functional differentiation. These 38 GATA genes are unevenly distributed across the 15 chromosomes of poplar, with tandem duplication events observed in two gene pairs and segmental duplication events occurring in 10 pairs of GATA genes. This suggests that gene duplication events have played a role in the expansion of the poplar GATA gene family, with segmental duplication events potentially serving as a primary driving force. Through gene collinearity analysis across species, 74 pairs of collinear gene pairs were identified, particularly within the fourth class of poplar GATA genes, which exhibit the same collinear relationship as multiple genes in monocotyledonous and dicotyledonous plants. This finding provides valuable insights for comparative genomics studies. By analyzing the expression patterns of these GATA genes, five key tissue-differential DEGs and eight salt-stress-responsive genes were identified. The characterization of GATA6, which is strongly induced by salt stress and is overlapped from two gene sets, revealed that it is a nuclear-localized protein with transcriptional activation activity. Additionally, *GATA6* is continuously induced by salt stress in the leaf and root tissues of poplar. Through analysis of the gene co-expression network centered around *GATA6*, it was found that these genes are likely involved in poplar growth, development, and abiotic stress response processes through cell transport systems and protein modification mechanisms.

## Data Availability

All data generated or analyzed during this study are included in this published article and its Supplementary Information files.

## Supplementary Materials

The supporting information can be downloaded at https://www.mdpi.com/article/10.3390/ijms241814118/s1.

## References

[1] Evans T., Reitman M., Felsenfeld G. (1988). An erythrocyte-specific DNA-binding factor recognizes a regulatory sequence common to all chicken globin genes. Proc. Natl. Acad. Sci. USA.

[2] Martin D.I., Tsai S.-F., Orkin S.H. (1989). Increased γ-globin expression in a nondeletion HPFH mediated by an erythroid-specific DNA-binding factor. Nature.

[3] Daniel-Vedele F., Caboche M. (1993). A tobacco cDNA clone encoding a GATA-1 zinc finger protein homologous to regulators of nitrogen metabolism in fungi. Mol. Genet. Genom..

[4] Behringer C., Schwechheimer C. (2015). B-GATA transcription factors—Insights into their structure, regulation, and role in plant development. Front. Plant Sci..

[5] Kim M., Xi H., Park J. (2021). Genome-wide comparative analyses of GATA transcription factors among 19 *Arabidopsis* ecotype genomes: Intraspecific characteristics of GATA transcription factors. PLoS ONE.

[6] Reyes J.C., Muro-Pastor M.I., Florencio F.J. (2004). The GATA Family of Transcription Factors in Arabidopsis and Rice. Plant Physiol..

[7] Manzoor M.A., Sabir I.A., Shah I.H., Wang H., Yu Z., Rasool F., Mazhar M.Z., Younas S., Abdullah M., Cai Y. (2021). Comprehensive Comparative Analysis of the GATA Transcription Factors in Four Rosaceae Species and Phytohormonal Response in Chinese Pear (*Pyrus bretschneideri*) Fruit. Int. J. Mol. Sci..

[8] Peng W., Li W., Song N., Tang Z., Liu J., Wang Y., Pan S., Dai L., Wang B. (2021). Genome-Wide Characterization, Evolution, and Expression Profile Analysis of GATA Transcription Factors in *Brachypodium distachyon*. Int. J. Mol. Sci..

[9] Schwechheimer C., Schröder P.M., Blaby-Haas C.E. (2022). Plant GATA Factors: Their Biology, Phylogeny, and Phylogenomics. Annu. Rev. Plant Biol..

[10] Teakle G.R., Manfield I.W., Graham J.F., Gilmartin P.M. (2002). *Arabidopsis thaliana* GATA factors: Organisation, expression and DNA-binding characteristics. Plant Mol. Biol..

[11] Luo X.-M., Lin W.-H., Zhu S., Zhu J.-Y., Sun Y., Fan X.-Y., Cheng M., Hao Y., Oh E., Tian M. (2010). Integration of Light- and Brassinosteroid-Signaling Pathways by a GATA Transcription Factor in *Arabidopsis*. Dev. Cell.

[12] Ravindran P., Verma V., Stamm P., Kumar P.P. (2017). A Novel RGL2–DOF6 Complex Contributes to Primary Seed Dormancy in *Arabidopsis thaliana* by Regulating a GATA Transcription Factor. Mol. Plant.

[13] Liu P.-P., Koizuka N., Martin R.C., Nonogaki H. (2005). The BME3 (Blue Micropylar End 3) GATA zinc finger transcription factor is a positive regulator of *Arabidopsis* seed germination. Plant J..

[14] Zhao Y., Medrano L., Ohashi K., Fletcher J.C., Yu H., Sakai H., Meyerowitz E.M. (2004). HANABA TARANU is a GATA tran-scription factor that regulates shoot apical meristem and flower development in *Arabidopsis*. Plant Cell.

[15] Shikata M., Matsuda Y., Ando K., Nishii A., Takemura M., Yokota A., Kohchi T. (2004). Characterization of *Arabidopsis* ZIM, a member of a novel plant-specific GATA factor gene family. J. Exp. Bot..

[16] Bi Y.-M., Zhang Y., Signorelli T., Zhao R., Zhu T., Rothstein S. (2005). Genetic analysis of *Arabidopsis* GATA transcription factor gene family reveals a nitrate-inducible member important for chlorophyll synthesis and glucose sensitivity. Plant J..

[17] An Y., Han X., Tang S., Xia X., Yin W. (2014). Poplar GATA transcription factor PdGNC is capable of regulating chloroplast ultrastructure, photosynthesis, and vegetative growth in *Arabidopsis* under varying nitrogen levels. Plant Cell Tissue Organ Cult..

[18] An Y., Zhou Y., Han X., Shen C., Wang S., Liu C., Yin W., Xia X. (2019). The GATA transcription factor GNC plays an important role in photosynthesis and growth in poplar. J. Exp. Bot..

[19] Richter R., Bastakis E., Schwechheimer C. (2013). Cross-Repressive Interactions between SOC1 and the GATAs GNC and GNL/CGA1 in the Control of Greening, Cold Tolerance, and Flowering Time in Arabidopsis. Plant Physiol..

[20] Lu G., Casaretto J.A., Ying S., Mahmood K., Liu F., Bi Y.-M., Rothstein S.J. (2017). Overexpression of OsGATA12 regulates chlorophyll content, delays plant senescence and improves rice yield under high density planting. Plant Mol. Biol..

[21] Yu Y.-H., Bian L., Yu K.-K., Yang S.-D., Zhang G.-H., Guo D.-L. (2020). Grape (*Vitis davidii*) VdGATA2 functions as a transcription activator and enhances powdery mildew resistance via the active oxygen species pathway. Sci. Hortic..

[22] Deb A., Kundu S. (2015). Deciphering Cis-Regulatory Element Mediated Combinatorial Regulation in Rice under Blast Infected Condition. PLoS ONE.

[23] Shen C., Zhang Y., Li Q., Liu S., He F., An Y., Zhou Y., Liu C., Yin W., Xia X. (2021). *PdGNC* confers drought tolerance by mediating stomatal closure resulting from NO and H_2_O_2_ production via the direct regulation of *PdHXK1* expression in *Populus*. New Phytol..

[24] Zhao T., Wu T., Pei T., Wang Z., Yang H., Jiang J., Zhang H., Chen X., Li J., Xu X. (2021). Overexpression of SlGATA17 Promotes Drought Tolerance in Transgenic Tomato Plants by Enhancing Activation of the Phenylpropanoid Biosynthetic Pathway. Front. Plant Sci..

[25] Nutan K.K., Singla-Pareek S.L., Pareek A. (2019). The Saltol QTL-localized transcription factor OsGATA8 plays an important role in stress tolerance and seed development in Arabidopsis and rice. J. Exp. Bot..

[26] Wang Y., Cao X., Zhang D., Li Y., Wang Q., Ma F., Xu X., Zhan X., Hu T. (2023). SlGATA17, A tomato GATA protein, interacts with SlHY5 to modulate salinity tolerance and germination. Environ. Exp. Bot..

[27] Zhu H., Zhai H., He S., Zhang H., Gao S., Liu Q. (2021). A novel sweetpotato GATA transcription factor, IbGATA24, interacting with IbCOP9-5a positively regulates drought and salt tolerance. Environ. Exp. Bot..

[28] Richter R., Behringer C., Müller I.K., Schwechheimer C. (2010). The GATA-type transcription factors GNC and GNL/CGA1 repress gibberellin signaling downstream from DELLA proteins and PHYTOCHROME-INTERACTING FACTORS. Genes Dev..

[29] Richter R., Behringer C., Zourelidou M., Schwechheimer C. (2013). Convergence of auxin and gibberellin signaling on the regulation of the GATA transcription factors *GNC* and *GNL* in *Arabidopsis thaliana*. Proc. Natl. Acad. Sci. USA.

[30] Ranftl Q.L., Bastakis E., Klermund C., Schwechheimer C. (2016). LLM-Domain Containing B-GATA Factors Control Different Aspects of Cytokinin-Regulated Development in *Arabidopsis thaliana*. Plant Physiol..

[31] Sen S., Kundu S., Dutta S.K. (2016). Proteomic analysis of JAZ interacting proteins under methyl jasmonate treatment in finger millet. Plant Physiol. Biochem..

[32] Shen C., Li Q., An Y., Zhou Y., Zhang Y., He F., Chen L., Liu C., Mao W., Wang X. (2022). The transcription factor GNC optimizes nitrogen use efficiency and growth by up-regulating the expression of nitrate uptake and assimilation genes in poplar. J. Exp. Bot..

[33] Liu M., Huang L., Zhang Y., Yan Z., Wang N. (2022). Overexpression of *PdeGATA3* results in a dwarf phenotype in poplar by promoting the expression of *PdeSTM* and altering the content of gibberellins. Tree Physiol..

[34] Ren M., Zhang Y., Liu C., Liu Y., Tian S., Cheng H., Zhang H., Wei H., Wei Z. (2021). Characterization of a High Hierarchical Regulator, PtrGATA12, Functioning in Differentially Regulating Secondary Wall Component Biosynthesis in *Populus trichocarpa*. Front. Plant Sci..

[35] Letunic I., Bork P. (2017). 20 years of the SMART protein domain annotation resource. Nucleic Acids Res..

[36] Paysan-Lafosse T., Blum M., Chuguransky S., Grego T., Pinto B.L., Salazar G.A., Bileschi M.L., Bork P., Bridge A., Colwell L. (2023). InterPro in 2022. Nucleic Acids Res..

[37] Zhao K., Dang H., Zhou L., Hu J., Jin X., Han Y., Wang S. (2023). Genome-Wide Identification and Expression Analysis of the HSF Gene Family in Poplar. Forests.

[38] Yu Z., Zhang F., Friml J., Ding Z. (2022). Auxin signaling: Research advances over the past 30 years. J. Integr. Plant Biol..

[39] Gao S., Chu C. (2020). Gibberellin Metabolism and Signaling: Targets for Improving Agronomic Performance of Crops. Plant Cell Physiol..

[40] Kishor P.B.K., Tiozon R.N., Fernie A.R., Sreenivasulu N. (2022). Abscisic acid and its role in the modulation of plant growth, development, and yield stability. Trends Plant Sci..

[41] Wang Y., Mostafa S., Zeng W., Jin B. (2021). Function and Mechanism of Jasmonic Acid in Plant Responses to Abiotic and Biotic Stresses. Int. J. Mol. Sci..

[42] Yao T., Song L., Xu W., DeMartino G.N., Florens L., Swanson S.K., Washburn M.P., Conaway R.C., Conaway J.W., Cohen R.E. (2006). Proteasome recruitment and activation of the Uch37 deubiquitinating enzyme by Adrm1. Nature.

[43] Plevin M.J., Mills M.M., Ikura M. (2005). The LxxLL motif: A multifunctional binding sequence in transcriptional regulation. Trends Biochem. Sci..

[44] Zhao X., Guo X., Tang X., Zhang H., Wang M., Kong Y., Zhang X., Zhao Z., Lv M., Li L. (2018). Misregulation of ER-Golgi Vesicle Transport Induces ER Stress and Affects Seed Vigor and Stress Response. Front. Plant Sci..

[45] Luo C., Shi Y., Xiang Y. (2022). SNAREs Regulate Vesicle Trafficking During Root Growth and Development. Front. Plant Sci..

[46] Xu F.-Q., Xue H.-W. (2019). The ubiquitin-proteasome system in plant responses to environments. Plant Cell Environ..

[47] Zientara-Rytter K., Sirko A. (2016). To deliver or to degrade—An interplay of the ubiquitin-proteasome system, autophagy and vesicular transport in plants. FEBS J..

[48] Jiang K., Yung V., Chiba T., Feldman L.J. (2017). Longitudinal patterning in roots: A GATA2–auxin interaction underlies and maintains the root transition domain. Planta.

[49] Tuskan G.A., DiFazio S., Jansson S., Bohlmann J., Grigoriev I., Hellsten U., Putnam N., Ralph S., Rombauts S., Salamov A. (2006). The Genome of Black Cottonwood, *Populus trichocarpa* (Torr. & Gray). Science.

[50] Goodstein D.M., Shu S., Howson R., Neupane R., Hayes R.D., Fazo J., Mitros T., Dirks W., Hellsten U., Putnam N. (2012). Phytozome: A comparative platform for green plant genomics. Nucleic Acids Res..

[51] Mistry J., Chuguransky S., Williams L., Qureshi M., Salazar G.A., Sonnhammer E.L.L., Tosatto S.C., Paladin L., Raj S., Richardson L.J. (2021). Pfam: The protein families database in 2021. Nucleic Acids Res..

[52] Lamesch P., Berardini T.Z., Li D., Swarbreck D., Wilks C., Sasidharan R., Muller R., Dreher K., Alexander D.L., Garcia-Hernandez M. (2011). The Arabidopsis Information Resource (TAIR): Improved gene annotation and new tools. Nucleic Acids Res..

[53] Chen C.J., Chen H., Zhang Y., Thomas H.R., Frank M.H., He Y.H., Xia R. (2020). TBtools: An Integrative Toolkit Developed for Interactive Analyses of Big Biological Data. Mol. Plant.

[54] Letunic I., Bork P. (2021). Interactive Tree Of Life (iTOL) v5: An online tool for phylogenetic tree display and annotation. Nucleic Acids Res..

[55] Hall T.A. (1999). Bioedit: A user-friendly biological sequence alignment editor and analysis program for windows 95/98/NT. Nucleic Acids Symp. Ser..

[56] Thompson J.D., Gibson T.J., Plewniak F., Jeanmougin F., Higgins D.G. (1997). The CLUSTAL_X windows interface: Flexible strategies for multiple sequence alignment aided by quality analysis tools. Nucleic Acids Res..

[57] Gasteiger E., Hoogland C., Gattiker A., Duvaud S.E., Wilkins M.R., Appel R.D., Bairoch A., Walker J.M. (2005). Protein Identification and Analysis Tools on the ExPASy Server. The Proteomics Protocols Handbook.

[58] Horton P., Park K.-J., Obayashi T., Fujita N., Harada H., Adams-Collier C.J., Nakai K. (2007). WoLF PSORT: Protein localization predictor. Nucleic Acids Res..

[59] Lescot M., Déhais P., Thijs G., Marchal K., Moreau Y., Van de Peer Y., Rouzé P., Rombauts S. (2002). PlantCARE, a database of plant cis-acting regulatory elements and a portal to tools for in silico analysis of promoter sequences. Nucleic Acids Res..

[60] Chow C.-N., Lee T.-Y., Hung Y.-C., Li G.-Z., Tseng K.-C., Liu Y.-H., Kuo P.-L., Zheng H.-Q., Chang W.-C. (2018). PlantPAN3.0: A new and updated resource for reconstructing transcriptional regulatory networks from ChIP-seq experiments in plants. Nucleic Acids Res..

[61] Hu B., Jin J., Guo A.-Y., Zhang H., Luo J., Gao G. (2015). GSDS 2.0: An upgraded gene feature visualization server. Bioinformatics.

[62] Bailey T.L., Boden M., Buske F.A., Frith M., Grant C.E., Clementi L., Ren J., Li W.W., Noble W.S. (2009). MEME SUITE: Tools for motif discovery and searching. Nucleic Acids Res..

[63] Wang Y., Tang H., DeBarry J.D., Tan X., Li J., Wang X., Lee T.-H., Jin H., Marler B., Guo H. (2012). *MCScanX*: A toolkit for detection and evolutionary analysis of gene synteny and collinearity. Nucleic Acids Res..

[64] Love M.I., Huber W., Anders S. (2014). Moderated estimation of fold change and dispersion for RNA-seq data with DESeq2. Genome Biol..

[65] Langfelder P., Horvath S. (2008). WGCNA: An R package for weighted correlation network analysis. BMC Bioinform..

[66] Shannon P., Markiel A., Ozier O., Baliga N.S., Wang J.T., Ramage D., Amin N., Schwikowski B., Ideker T. (2003). Cytoscape: A software environment for integrated models of Biomolecular Interaction Networks. Genome Res..

