# Supplementary material for: Comprehensive Analysis and Characterization of the GATA Gene Family, with Emphasis on the GATA6 Transcription Factor in Poplar
Source: Int J Mol Sci. 2023 Sep 14;24(18):14118. doi: 10.3390/ijms241814118 (PMC10532138; doi:10.3390/ijms241814118)
Supplement: Supplementary file 1 [file ijms-24-14118-s001.zip › Supplemental Table S1.pdf]

Table S1: Accession numbers, physicochemical properties, and subcellular localization predictions of poplar GATA family members.

Gene Name	Accession number	AA[a]	MW[b] (Da)	pI[c]	II[d]	AI[e]	GRAVY[f]	Subcellular Localization
PtrGATA1	Potri.001G053500.2	258	28925.58	8.55	53.49	61.59	-0.755	Nucleus
PtrGATA2	Potri.001G151700.2	544	60287.6	6.77	50.78	72.74	-0.644	Chloroplast
PtrGATA3	Potri.001G188500.3	303	33611.21	5.81	58.46	49.93	-0.706	Nucleus
PtrGATA4	Potri.002G110800.1	360	39264.4	4.86	47.96	69.33	-0.702	Nucleus
PtrGATA5	Potri.002G110900.1	290	31499.81	5.93	45.45	60.83	-0.698	Nucleus
PtrGATA6	Potri.002G142800.1	246	27390.13	6.03	52.06	55.89	-0.738	Nucleus
PtrGATA7	Potri.002G199800.1	147	16627.16	9.4	65.96	54.49	-0.879	Nucleus
PtrGATA8	Potri.003G082800.1	540	60284.54	6.58	54.94	70.57	-0.672	Chloroplast
PtrGATA9	Potri.003G174800.2	258	28865.61	9.06	52.17	61.2	-0.783	Nucleus
PtrGATA10	Potri.003G213300.1	226	25422.98	8.74	38.45	45	-1.071	Nucleus
PtrGATA11	Potri.004G161500.1	327	36312.44	8.53	56.91	50.4	-0.73	Nucleus
PtrGATA12	Potri.004G211800.1	301	33982.35	8.57	68.69	56.38	-0.674	Nucleus
PtrGATA13	Potri.005G020500.1	161	17587.24	9.78	51.5	60	-0.675	Nucleus
PtrGATA14	Potri.005G066100.2	240	27608.63	8.71	59.12	68.75	-0.58	Nucleus
PtrGATA15	Potri.005G117600.1	333	36840.45	7.1	62.45	60.54	-0.63	Nucleus
PtrGATA16	Potri.005G122700.1	259	29015.8	7.95	64.92	35.48	-0.973	Nucleus
PtrGATA17	Potri.005G152500.1	365	39708.23	5.13	43.65	67.84	-0.626	Nucleus
PtrGATA18	Potri.005G152800.1	288	31463.7	5.67	44.14	61.94	-0.698	Nucleus
PtrGATA19	Potri.006G229200.3	303	33567.46	9.04	59.98	56.4	-0.772	Nucleus
PtrGATA20	Potri.006G237700.1	373	41397.57	6.04	45.33	56.43	-0.791	Nucleus
PtrGATA21	Potri.007G016600.1	376	41808.29	6.95	64.79	57.5	-0.589	Chloroplast
PtrGATA22	Potri.007G024500.1	254	28668.6	8.46	64.84	38.07	-0.941	Nucleus
PtrGATA23	Potri.007G116550.1	318	33403.12	5.49	34.43	66.92	-0.462	Extracellular
PtrGATA24	Potri.007G116700.1	384	43220.87	4.9	43.09	58.1	-0.852	Nucleus
PtrGATA25	Potri.008G038900.2	354	38941.87	6.52	56.02	60.31	-0.597	Nucleus
PtrGATA26	Potri.008G213900.1	138	14783.7	9.69	61.85	63.04	-0.909	Nucleus
PtrGATA27	Potri.009G123400.1	329	36593.74	6.61	52.09	55.99	-0.629	Nucleus
PtrGATA28	Potri.010G001300.2	149	16059.17	9.68	63.22	64.9	-0.87	Nucleus
PtrGATA29	Potri.010G223300.2	352	38612.24	5.65	55.82	57.33	-0.591	Nucleus
PtrGATA30	Potri.010G251600.1	307	33369.72	7.8	29.88	48.96	-0.893	Nucleus
PtrGATA31	Potri.013G059600.1	295	32644.49	6.53	51.49	61.15	-0.754	Nucleus
PtrGATA32	Potri.014G058600.1	251	27963.8	6.42	60.52	57.49	-0.78	Nucleus
PtrGATA33	Potri.014G124400.1	133	14704.83	9.77	67.48	60.98	-0.841	Nucleus
PtrGATA34	Potri.017G042200.2	383	43298.04	5.11	44.15	59.01	-0.864	Nucleus
PtrGATA35	Potri.017G042300.3	109	12596.12	10.69	40.81	84.95	-0.134	Cytoplasmic
PtrGATA36	Potri.018G044900.1	380	42096.26	6.22	41.9	56.18	-0.802	Nucleus
PtrGATA37	Potri.018G053600.1	303	33666.66	8.78	50.69	60.83	-0.74	Nucleus
PtrGATA38	Potri.019G033000.1	294	32231.74	6	51.37	56.43	-0.792	Nucleus

[a] Length of the amino acid sequence; [b] Molecular weight of the protein; [c] Theoretical pI of the protein; [d] Instability index (II) of the protein; [e] Aliphatic index of the protein; [f] Grand average of hydropathicity of the protein.
