# Supplementary material for: Comprehensive Analysis and Characterization of the GATA Gene Family, with Emphasis on the GATA6 Transcription Factor in Poplar
Source: Int J Mol Sci. 2023 Sep 14;24(18):14118. doi: 10.3390/ijms241814118 (PMC10532138; doi:10.3390/ijms241814118)
Supplement: Supplementary file 1 [file ijms-24-14118-s001.zip › Supplemental Table S2.pdf]

Table S2: Motifs and their annotation information in poplar GATA proteins.

Annotations of GATA protein sequence motifs

Name	Sequence	Description of Smart	Description of Pfam
Motif 1	HCGTTKTPQWRRGPAGPKTLCNACGVRYK	NO	GATA
Motif 2	GRLVPEYRPAASPTFVSELHSNSHRKVLE	NO	NO
Motif 3	RSTQPRRIASLVRFREKRKERCFDKKIRYTVRKEVALRMHRKKGQFASSK	NO	CCT
Motif 4	ELCVPTDDIAELEWLSNFVEDSFSDYSL	NO	NO
Motif 5	EDGVVPSSRTSZLTLSFEGEVYVFPAVTPDKVQAVLLLLGGREIPTA	TIFY	tify
Motif 6	SVSGSDYFSVDDLLDFSNDDDDAEDTD	NO	NO
Motif 7	TRVPGKARSKRSRAAPRTWR	NO	NO
Motif 8	PELRKRKLGEEEQAAILLMALSCGSVYA	NO	NO
Motif 9	KKEMVRAQRQKQHRKGRDHHNRMVFQIEGGGDDYGYGFDDG	low complexity	NO
Motif 10	NKGTLRDLSKGGRNJPFNQNEPVTPDFKP	NO	NO
Motif 11	MMHRCSSSQCNMVGPCSCGMYHSQSNSFSMLFSMPDHRKSFDETDMYP	NO	NO
Motif 12	MEQHYGYHQNNSWVQNQKMPCFSPANEFRFIEDNDRDSDTG	NO	NO
Motif 13	MYTPSQPMNVHNQIVSPGVDDDGAPADPIDHHHHIHYEDGTPAVV	low complexity	NO
Motif 14	FEEFARHLTNEEQQQLLKYLPPLDTAKLPNSIESMFDSPQFKENINCYQQ	NO	UCH-binding
Motif 15	VKWMPSKMRLMQEMTNSNCSETDHQPMKFMLKFHNQQCQNNEIN	NO	NO
Motif 16	VEKLTKDLYTILHEQQSSCFSGSSEEDLLFDNETPMVSVEIGHGSVLIRH	NO	NO
Motif 17	PFAEEELEWLLDDDDFPNVLDCFGDLSGEPGEIPEHHDPQVVJEDSST	low complexity	NO
Motif 18	GTPSTRLSEDDEKRMRHDQRRSGSCMSNFCWDILQTKNDSTPYPPQAHKT	NO	NO
Motif 19	FAESRLVIKIPEHVDPEHKKKKKIKFIVPLGPVEMNQNSQPQQAVRKCM	low complexity	NO
Motif 20	MRRQKEMLGPEQQQ	NO	NO
