# Supplementary material for: Comprehensive Analysis and Characterization of the GATA Gene Family, with Emphasis on the GATA6 Transcription Factor in Poplar
Source: Int J Mol Sci. 2023 Sep 14;24(18):14118. doi: 10.3390/ijms241814118 (PMC10532138; doi:10.3390/ijms241814118)
Supplement: Supplementary file 1 [file ijms-24-14118-s001.zip › Supplemental Table S3.pdf]

Table S3: Tandemly or segmentally duplicated poplar *GATA* gene pairs.

Name1	Name2	Duplication Type	Ka	Ks	Ka/Ks	Selection Pressure
PtrGATA4	PtrGATA5	tandem duplication	0.429698813	1.855869185	0.231535076	Purifying selection
PtrGATA34	PtrGATA35	tandem duplication	0.426080913	NaN	NaN	No
PtrGATA1	PtrGATA9	Segmental duplication	0.057473093	0.316440172	0.181623883	Purifying selection
PtrGATA15	PtrGATA21	Segmental duplication	0.087057492	0.353709629	0.246127005	Purifying selection
PtrGATA16	PtrGATA22	Segmental duplication	0.044386501	0.213230334	0.208162225	Purifying selection
PtrGATA2	PtrGATA8	Segmental duplication	0.037638274	0.165342398	0.227638372	Purifying selection
PtrGATA20	PtrGATA36	Segmental duplication	0.044042799	0.255510416	0.172371835	Purifying selection
PtrGATA23	PtrGATA35	Segmental duplication	0.278797311	0.718673014	0.387933463	Purifying selection
PtrGATA24	PtrGATA34	Segmental duplication	0.0716354	0.287947801	0.248779118	Purifying selection
PtrGATA31	PtrGATA38	Segmental duplication	0.106485544	0.251183725	0.423934886	Purifying selection
PtrGATA6	PtrGATA32	Segmental duplication	0.062784223	0.299724621	0.209473024	Purifying selection
PtrGATA7	PtrGATA33	Segmental duplication	0.088162344	0.228158531	0.386408273	Purifying selection
