# Supplementary material for: Comprehensive Analysis and Characterization of the GATA Gene Family, with Emphasis on the GATA6 Transcription Factor in Poplar
Source: Int J Mol Sci. 2023 Sep 14;24(18):14118. doi: 10.3390/ijms241814118 (PMC10532138; doi:10.3390/ijms241814118)
Supplement: Supplementary file 1 [file ijms-24-14118-s001.zip › Supplemental Table S4.pdf]

Table S4: Syntenic gene pairs among different species.

Syntenic gene pairs (*P. trichocarpa* and *A. thaliana*)

Gene ID	Gene ID	Ka	Ks	Ka/Ks	Selection Pressure
PtrGATA2	AT5G47140.1	0.303278967	1.648740351	0.183945863	Purifying selection
PtrGATA6	AT2G45050.1	0.283918241	3.711569918	0.076495458	Purifying selection
PtrGATA6	AT3G60530.1	0.263736761	1.942831482	0.135748655	Purifying selection
PtrGATA8	AT4G17570.2	0.292931563	1.375292751	0.212995788	Purifying selection
PtrGATA16	AT2G18380.1	0.432558889	2.137317779	0.202383985	Purifying selection
PtrGATA15	AT3G51080.1	0.395179718	NaN	NaN	No
PtrGATA15	AT4G36240.1	0.426044017	2.72818962	0.156163638	Purifying selection
PtrGATA16	AT4G36620.1	0.361456378	3.171843881	0.113957809	Purifying selection
PtrGATA15	AT5G66320.1	0.365724168	NaN	NaN	No
PtrGATA22	AT2G18380.1	0.444545711	4.348344832	0.102233316	Purifying selection
PtrGATA21	AT4G36240.1	0.423636434	3.550211351	0.119327103	Purifying selection
PtrGATA22	AT4G36620.1	0.32400422	2.08953503	0.155060439	Purifying selection

Syntenic gene pairs (*P. trichocarpa* and *G. max*)

Gene ID	Gene ID	Ka	Ks	Ka/Ks	Selection Pressure
PtrGATA2	Glyma.01G205100.1	0.200346494	0.996536227	0.20104286	Purifying selection
PtrGATA2	Glyma.05G064800.1	0.195631192	1.171416534	0.167003953	Purifying selection
PtrGATA3	Glyma.07G016800.1	0.295779126	NaN	NaN	No
PtrGATA3	Glyma.08G202100.1	0.326257225	NaN	NaN	No
PtrGATA2	Glyma.11G037900.1	0.190874083	0.965761179	0.197641081	Purifying selection
PtrGATA2	Glyma.17G146600.1	0.20063993	1.118888077	0.179320823	Purifying selection
PtrGATA8	Glyma.01G205100.1	0.20237644	1.125558189	0.179800957	Purifying selection
PtrGATA8	Glyma.05G064800.1	0.197330672	1.261515864	0.156423456	Purifying selection
PtrGATA8	Glyma.11G037900.1	0.201536009	1.029605176	0.195741061	Purifying selection
PtrGATA8	Glyma.17G146600.1	0.199067701	1.245768961	0.15979504	Purifying selection
PtrGATA15	Glyma.01G169400.1	0.260271777	4.235714281	0.061446963	Purifying selection

PtrGATA15	Glyma.02G051100.1	0.284523184	NaN	NaN	No
PtrGATA16	Glyma.02G056600.1	0.263452843	NaN	NaN	No
PtrGATA18	Glyma.02G215600.1	0.231009125	0.850379474	0.271654163	Purifying selection
PtrGATA13	Glyma.03G232900.1	0.181251969	NaN	NaN	No
PtrGATA16	Glyma.11G068700.1	0.201497968	NaN	NaN	No
PtrGATA18	Glyma.14G182800.1	0.215705964	0.935351378	0.230614899	Purifying selection
PtrGATA16	Glyma.16G139400.1	0.259701979	NaN	NaN	No
PtrGATA13	Glyma.19G229900.1	0.275841253	NaN	NaN	No
PtrGATA19	Glyma.04G051300.1	0.434389023	1.51657945	0.286426816	Purifying selection
PtrGATA20	Glyma.04G084900.1	0.230465301	4.964454665	0.046423085	Purifying selection
PtrGATA20	Glyma.06G086400.1	0.253430218	4.547196036	0.055733295	Purifying selection
PtrGATA19	Glyma.14G094800.1	0.431548358	1.716668824	0.251387077	Purifying selection
PtrGATA19	Glyma.17G228700.1	0.475806273	1.880534629	0.253016491	Purifying selection
PtrGATA21	Glyma.01G169400.1	0.286719009	1.934885409	0.148183974	Purifying selection
PtrGATA21	Glyma.02G051100.1	0.305336913	NaN	NaN	No
PtrGATA22	Glyma.02G056600.1	0.255027024	NaN	NaN	No
PtrGATA22	Glyma.11G068700.1	0.213393233	NaN	NaN	No
PtrGATA22	Glyma.16G139400.1	0.258204228	NaN	NaN	No
PtrGATA25	Glyma.10G210500.1	0.216925925	1.704992941	0.127229808	Purifying selection
PtrGATA25	Glyma.20G180100.1	0.218028979	1.934194116	0.112723422	Purifying selection
PtrGATA31	Glyma.16G042300.1	0.291864937	1.597028156	0.182755035	Purifying selection
PtrGATA31	Glyma.19G110200.1	0.25354121	1.824337162	0.138977167	Purifying selection
PtrGATA34	Glyma.08G067200.1	0.341750994	1.391529122	0.24559385	Purifying selection
PtrGATA36	Glyma.04G084900.1	0.262522715	2.126516219	0.123452017	Purifying selection
PtrGATA36	Glyma.06G086400.1	0.282367964	1.726659958	0.163534206	Purifying selection
PtrGATA38	Glyma.16G042300.1	0.29710064	1.381667455	0.215030497	Purifying selection
PtrGATA38	Glyma.19G110200.1	0.262299742	1.545926683	0.169671528	Purifying selection

Syntenic gene pairs (*P. trichocarpa* and *S. lycopersicum*)

Gene ID	Gene ID	Ka	Ks	Ka/Ks	Selection Pressure
PtrGATA3	Solyc03g120890.2.1	0.382505219	2.6973796	0.141806225	Purifying selection
PtrGATA2	Solyc08g077960.2.1	0.221705389	1.431282855	0.154899773	Purifying selection
PtrGATA2	Solyc08g007190.2.1	0.216415281	1.401711831	0.154393561	Purifying selection
PtrGATA6	Solyc01g090760.2.1	0.267127866	2.17509595	0.122811992	Purifying selection
PtrGATA7	Solyc01g100220.2.1	0.461838444	1.740293784	0.265379586	Purifying selection
PtrGATA4	Solyc04g076530.2.1	0.268489503	1.456690378	0.184314737	Purifying selection
PtrGATA7	Solyc09g075610.2.1	0.267686909	1.417048454	0.188904556	Purifying selection
PtrGATA8	Solyc08g077960.2.1	0.206010393	1.312857059	0.156917611	Purifying selection
PtrGATA13	Solyc01g100220.2.1	0.388522876	3.954749669	0.098242091	Purifying selection
PtrGATA15	Solyc02g084590.2.1	0.299467471	2.572708943	0.116401613	Purifying selection
PtrGATA16	Solyc02g085190.1.1	0.21980105	1.158854163	0.189671019	Purifying selection
PtrGATA15	Solyc02g062380.1.1	0.400207395	3.56611572	0.112225016	Purifying selection
PtrGATA16	Solyc02g062760.2.1	0.309459826	NaN	NaN	No
PtrGATA15	Solyc03g033660.2.1	0.389136207	NaN	NaN	No
PtrGATA17	Solyc04g076530.2.1	0.296003079	1.38773021	0.213300162	Purifying selection
PtrGATA20	Solyc08g066510.2.1	0.293869138	2.542457214	0.115584694	Purifying selection
PtrGATA21	Solyc02g084590.2.1	0.308119562	2.285749394	0.134800238	Purifying selection
PtrGATA22	Solyc02g085190.1.1	0.21475443	1.47286008	0.145807761	Purifying selection
PtrGATA33	Solyc09g075610.2.1	0.310045034	1.988772948	0.155897652	Purifying selection
PtrGATA34	Solyc01g106030.2.1	0.553949968	2.797981637	0.197981988	Purifying selection

Syntenic gene pairs (*P. trichocarpa* and *A. comosus*)

Gene ID	Gene ID	Ka	Ks	Ka/Ks	Selection Pressure
PtrGATA2	Aco012875.1	0.394040903	2.385477524	0.165183238	Purifying selection
PtrGATA2	Aco011659.1	0.329956271	1.973963143	0.167154221	Purifying selection
PtrGATA8	Aco012875.1	0.404094343	2.480913903	0.162881244	Purifying selection
PtrGATA8	Aco011659.1	0.326611184	1.678335346	0.194604246	Purifying selection

Syntenic gene pairs (*P. trichocarpa* and *O. sativa*)

Gene ID	Gene ID	Ka	Ks	Ka/Ks	Selection Pressure
NO					

Syntenic gene pairs (*P. trichocarpa* and *S. bicolor*)

Gene ID	Gene ID	Ka	Ks	Ka/Ks	Selection Pressure
NO					
