# Supplementary material for: Comprehensive Analysis and Characterization of the GATA Gene Family, with Emphasis on the GATA6 Transcription Factor in Poplar
Source: Int J Mol Sci. 2023 Sep 14;24(18):14118. doi: 10.3390/ijms241814118 (PMC10532138; doi:10.3390/ijms241814118)
Supplement: Supplementary file 1 [file ijms-24-14118-s001.zip › Supplemental Table S5.pdf]

Table S5: Primers used in this study.

Primer sequences

**Name**	**5′ Primers**	**3′ Primers**
GATA6_ gene clone	CTATGCATCCTTCATCTCCAAG	CTGCACCGCTGCACTACTAATC
GATA6_pBI121-GFP	GGAGAGAACACGGGGGACATGAATGTCTATGGAGGAG	CCTCGCCCTTGCTCACCATGCAAACTTCATAACC
GATA6_ pGBKT7	GCCATGGAGGCCGAATTCATGAATGTCTATGGAGGAG	TGCGGCCGCTGCAGGTCGTTAGCAAACTTCATAACC
GATA6_RT-qPCR	GACTTCTCTAATGATGACCTTC	CGATAGCCACTCTAGCTCTGCC
ACT	ACCCTCCAATCCAGACACTG	TTGCTGACCGTATGAGCAAG
